# Blood Pressure Variability Indices for Outcome Prediction After Thrombectomy in Stroke by Using High-Resolution Data

**DOI:** 10.1007/s12028-022-01519-x

**Published:** 2022-05-23

**Authors:** Corinne Inauen, Jens M. Boss, Mira Katan, Andreas R. Luft, Zsolt Kulcsar, Jan F. Willms, Stefan Y. Bögli, Emanuela Keller

**Affiliations:** 1 Neurocritical Care Unit, Department of Neurosurgery, Institution of Intensive Care Medicine, University Hospital Zurich, Zurich, Switzerland; 2 Department of Neurology, University Hospital Zurich, Zurich, Switzerland; 3 Clinical Neuroscience Center, University Hospital Zurich, University of Zurich, Zurich, Switzerland; 4 Cereneo Center for Neurology and Rehabilitation, Vitznau, Switzerland; 5 Department of Neuroradiology, University Hospital Zurich, Zurich, Switzerland

**Keywords:** Big data, Blood pressure, Critical care, Ischemic stroke, Thrombectomy

## Abstract

**Background:**

Blood pressure variability (BPV) is associated with outcome after endovascular thrombectomy in acute large vessel occlusion stroke. We aimed to provide the optimal sampling frequency and BPV index for outcome prediction by using high-resolution blood pressure (BP) data.

**Methods:**

Patient characteristics, 3-month outcome, and BP values measured intraarterially at 1 Hz for up to 24 h were extracted from 34 patients treated at a tertiary care center neurocritical care unit. Outcome was dichotomized (modified Rankin Scale 0–2, favorable, and 3–6, unfavorable) and associated with systolic BPV (as calculated by using standard deviation, coefficient of variation, averaged real variability, successive variation, number of trend changes, and a spectral approach using the power of specific BP frequencies). BP values were downsampled by either averaging or omitting all BP values within each prespecified time bin to compare the different sampling rates.

**Results:**

Out of 34 patients (age 72 ± 12.7 years, 67.6% men), 10 (29.4%) achieved a favorable functional outcome and 24 (70.6%) had an unfavorable functional outcome at 3 months. No group differences were found in mean absolute systolic BP (SBP) (130 ± 18 mm Hg, *p* = 0.82) and diastolic BP (DBP) (59 ± 10 mm Hg, *p* = 1.00) during the monitoring time. BPV only reached predictive significance when using successive variation extracted from downsampled (averaged over 5 min) SBP data (median 4.8 mm Hg [range 3.8–7.1]) in patients with favorable versus 7.1 mmHg [range 5.5–9.7] in those with unfavorable outcome, area under the curve = 0.74 [confidence interval (CI) 0.57–0.85; *p* = 0.031], or the power of midrange frequencies between 1/20 and 1/5 min [area under the curve = 0.75 (CI 0.59–0.86), *p* = 0.020].

**Conclusions:**

Using high-resolution BP data of 1 Hz, downsampling by averaging all BP values within 5-min intervals is essential to find relevant differences in systolic BPV, as noise can be avoided (confirmed by the significance of the power of midrange frequencies). These results demonstrate how high-resolution BP data can be processed for effective outcome prediction.

**Supplementary Information:**

The online version contains supplementary material available at 10.1007/s12028-022-01519-x.

## Introduction

Increased blood pressure variability (BPV) has been associated with unfavorable outcome in acute ischemic stroke (AIS) [1] and intracerebral hemorrhage (ICH) [2–6]. AIS leads to impaired autoregulation of cerebral blood flow and, therefore, to a higher vulnerability to BPV [7, 8]. This is particularly true in AIS due to large vessel occlusion (LVO) that leads to large lesions with focally impaired autoregulation [9, 10]. Various reasons for the effect of BPV on outcome have been proposed. Most prominently, the loss of potentially viable penumbral tissue due to low blood pressure (BP), or reperfusion injury and hemorrhagic transformation due to elevated BP have been discussed [1, 6]. Recently, it was shown that in patients with AIS-LVO treated with endovascular thrombectomy (EVT), the association of high BPV with poor outcome mainly affected patients with complete recanalization [11] or good collaterals [12]. Therefore, these patients are an ideal cohort for studying the effect of BPV on stroke outcome.

A variety of indices reflecting BPV (standard deviation [SD], coefficient of variation [CV], averaged real variability [ARV], successive variation [SV] as well as the relative number of trend changes) have been proposed as predictive for outcome in various cerebrovascular [6, 11], cardiovascular [13], and other conditions with dynamic vital signs, e.g., sepsis [14]. However, the considerable variability in experimental design (especially regarding the frequency of BP measurements) and analytical approach does not allow for direct comparison between findings, and thus prevents the possibility to draw conclusions regarding the optimal index and the optimal BP sampling frequency for outcome prediction in patients with AIS-LVO. So far, BPV for AIS outcome prediction was recorded at temporal resolutions of up to beat-to-beat (and if so for only up to 10-min recordings) when considering all stroke etiologies [4, 7, 15] and temporal resolutions of up to 1/15 min when specifically evaluating outcome after AIS-LVO treated with EVT [2, 6, 11]. Thus, rapid changes of BP remain undetected.

This study aims to present an analysis of different BPV indices using intraarterial BP measurements sampled at 1 Hz for up to 24 h to provide the optimal sampling frequency and BPV index for outcome prediction by using high-resolution BP data.

## Methods

The data are available on reasonable request by the corresponding author.

### Study Design and Patient Selection

We retrospectively reviewed data from all consecutive patients with an arterial line admitted to the neurocritical care unit at the University Hospital Zurich after EVT due to AIS-LVO from January 2017 to October 2020. Patients extubated after EVT were treated at the stroke unit and were excluded from the analysis. This study, which is part of the project “ICU Cockpit,” was approved by the local ethics committee. Written consent was given by the patients or by their legal representatives. Stroke diagnosis and cause was determined by using standardized diagnostic workup. Diagnostic workup prior to EVT consisted of computed tomography imaging including perfusion measurement and angiography. Further diagnostic workup included (at least) magnetic resonance imaging 24 h after AIS-LVO, a 12-channel electrocardiogram, 24-h BP monitoring, 48-h Holter monitoring, and a cerebrovascular ultrasound to evaluate the origin of AIS-LVO and the reperfusion/collateralization status [16]. Outcome was extracted from routine poststroke follow-up consultations at 3 months in the outpatient clinic. Patients were dichotomized based on their modified Rankin Scale (mRS). The primary outcome was defined as unfavorable functional outcome (90-day mRS of 3–6).

BP management during the stay at the intensive care unit (ICU) was guided by current American Heart Association/American Stroke Association guidelines [16, 17]. After intravenous tissue plasminogen activator administration, BP was targeted to values < 180/105 mm Hg. BP limits after EVT were defined for each case individually, as supported by the consensus decisions of the American Heart Association/American Stroke Association and recent studies [18] depending on recanalization status (< 160/90 and < 140/90 mm Hg after successful recanalization). The two patients with incomplete recanalization received a more liberal SBP target of 120–180 mm Hg.

### Data Collection

At ICU admission, patients had already received an arterial line for continuous BP monitoring via the standard monitoring system Phillips Intellivue (Philips Medical Systems, Boeblingen, Germany). Diastolic and SBP values were recorded continuously at a sampling rate of 1 Hz. Data from the Phillips systems were collected through a CNS data collector (Moberg ICU Solutions, Ambler, PA). For this study, we considered data from the first 24 h after thrombectomy. The high-resolution BP data were stored on a dedicated information technology infrastructure called the “ICU Cockpit.”

### BPV

BPV was elucidated by using different BPV indices and sampling frequencies (time domain) and a spectral approach.

### Indices

We calculated BPV for SBP and DBP by using five indices based on previous studies [3, 4, 6, 14]: SD, CV, ARV, SV, and trend changes using the following formulas:

$${\text{SD}}\left( {{\text{BP}}} \right) = \sqrt {\frac{1}{n - 1}\mathop \sum \limits_{i = 1}^{n} \left( {{\text{BP}}_{i} - {\text{mean}}\left( {{\text{BP}}} \right)} \right)^{2} } ,$$

$${\text{CV}}\left( {{\text{BP}}} \right) = \frac{{{\text{SD}}\left( {{\text{BP}}} \right)}}{{{\text{mean}}\left( {{\text{BP}}} \right)}},$$

$${\text{ARV}}\left( {{\text{BP}}} \right) = \frac{1}{n - 1}\mathop \sum \limits_{i = 1}^{n - 1} \left| {{\text{BP}}_{i + 1} - {\text{BP}}_{i} } \right| ,$$

$${\text{SV}}\left( {{\text{BP}}} \right) = \sqrt {\frac{1}{n - 1}\mathop \sum \limits_{i = 1}^{n - 1} \left| {{\text{BP}}_{i + 1} - {\text{BP}}_{i} } \right|^{2} } ,$$

$${\text{TC}}\left( {{\text{BP}}} \right) = \frac{{\# \left[ {\left( {{\text{BP}}_{i + 1} - {\text{BP}}_{i} } \right)*\left( {{\text{BP}}_{i + 2} - {\text{BP}}_{i + 1} } \right) < 0;\;\;i = 1,2, \ldots ,n - 2} \right]}}{n},$$

where ${\text{BP}} = \left( {{\text{BP}}_{1} ,{\text{BP}}_{2} , \ldots , {\text{BP}}_{n} } \right)$ is the series of either the consecutive DBP or SBP measurements, and *n* is the number of samples. The “#” indicates the number of cases corresponding with the expression in the brackets. Additionally, because not all patients had a full 24-h record, the number of available BP measurements was also included in the analysis.

### Time Domain

To investigate the effect of different sampling rates, we calculated the mentioned indices using sampling rates ranging from 1/1 s to 1/30 min. Two different methods were used to downsample the BP data: (1) to mimic a situation in which only the instantaneous BP values are known, we simply omitted all measurements between the respective timestamps (“instantaneous sampling method”). (2) by averaging all BP values within the respective time bins to achieve the mentioned sampling rate, thereby taking advantage of the high-resolution data (“averaging method”).

### Power Spectrum

To calculate BPV using Fourier Transformation [19–21], we defined four BP frequency bands: (1) < 1/20 min (low), (2) 1/20 min to 1/5 min (midrange), (3) 1/5 min to 1/1 min (high), (4) > 1/1 min (very high). The formula used to compute the power of these defined frequency bands was:

$$P = \mathop \sum \limits_{{k = k_{{{\text{start}}}} }}^{{k_{{{\text{end}}}} }} \frac{1}{{N^{2} }}\left| {\widehat{{{\text{BP}}}}_{k} } \right|^{2} = \mathop \sum \limits_{{k = k_{{{\text{start}}}} }}^{{k_{{{\text{end}}}} }} \left( {\frac{{t_{s} }}{N}\left| {\widehat{{{\text{BP}}}}_{k} } \right|^{2} } \right) \cdot {\Delta }f,$$

where $\widehat{{{\text{BP}}_{k} }}$ are the Fourier components of the BP, and $k_{{{\text{start}}}}$/$k_{{{\text{end}}}}$ are chosen so that the corresponding frequencies lie within the respective frequency band, i.e., $f_{k} \in \left[ {f_{{{\text{start}},}} f_{{{\text{end}}}} } \right) \forall k = k_{{{\text{start}}}} , \ldots , k_{{{\text{end}}}}$.

### Data Preprocessing

The unprocessed BP samples measured at 1 Hz exhibited artifacts and gaps due to patient manipulation and/or measurement error as well as monitoring intermittency. To remove artifacts, we used a threshold filter that compared the measured BP values to the moving average and removed data points that deviated more than 30 mm Hg from this average. To obtain the SV and ARV indices, we further processed the calculated differences between consecutive BP measurements to avoid oversensitivity to potential BP jumps due to artifact removal. For each patient, we computed the SD for the differences and masked values that exceeded 3*SD. Moreover, linear interpolation was used to fill these gaps before computing the power of frequency bands.

### Statistical Analysis

Statistical analysis was performed using SPSS (version 25). Descriptive statistics are reported as counts/percentages, mean ± SD, or as median (interquartile range [IQR]). Categorical variables are compared with Fisher’s exact test, and continuous/ordinal variables with Mann–Whitney *U*-test. A *p* value < 0.05 was considered statistically significant. Effect size is reported as the area under the receiver operating characteristics curve including the 95% confidence intervals (CI) [22]. BP data preprocessing and indices computation was performed using Python 3.8.

## Results

Thirty-four patients after EVT due to LVO stroke were included. Patient characteristics are summarized in Table 1. The average age was 72 ± 12.7 years, 23 (67.6%) were men. The median National Institutes of Health Stroke Scale (NIHSS) score on admission was 14 (IQR 8–19). Twenty-six (76.5%) underwent direct EVT, whereas eight (23.5%) received combined IV thrombolysis. Successful recanalization (defined as thrombolysis in cerebral ischemia 2b/3) was achieved in 32 (94.1%) cases. The time between symptom onset or last proof of good health to groin puncture did not differ between the groups (median 257 min [IQR 181–481]). There was a total of five outliers, three in the favorable and two in the unfavorable group. This can primarily be explained by the inclusion of wake-up strokes or later indication for EVT in case of secondary deterioration.

**Table 1:** Patient characteristics of the total cohort and comparison between patients with favorable (mRS 0–2) versus unfavorable outcome (mRS 3–6) at 3 months

	Total, *N* = 34 (100%)	Favorable, *n* = 10 (29.4%)	Unfavorable, *n* = 24 (70.6%)	*p* value
Demographic data				
Age, mean ± SD (yr)	72 ± 12.7	64 ± 10.9	74.9 ± 12.4	0.025
Male sex, *n* (%)	23 (67.6)	9 (90)	14 (58.3)	0.113
Medical history, n (%)				
Hypertension	25 (73.5)	10 (100)	15 (62.5)	0.034
Diabetes mellitus	7 (20.6)	2 (20)	5 (20.8)	1
Dyslipidaemia	13 (38.2)	5 (50)	8 (33.3)	0.451
Smoking	7 (20.6)	3 (30)	4 (16.7)	0.394
Previous stroke/TIA	5 (14.7)	0 (0)	5 (20.8)	0.291
Coronary heart disease	8 (23.5)	4 (40)	4 (16.7)	0.195
Atrial fibrillation	12 (35.3)	3 (30)	9 (37.5)	1
Medication history, *n* (%)				
Antiplatelet therapy	11 (32.4)	4 (40)	7 (29.2)	0.692
Anticoagulation	6 (17.6)	1 (10)	5 (20.8)	0.644
Antihypertensive therapy	16 (47.1)	6 (60)	10 (41.7)	0.457
Blood pressure at admission				
SBP, mean ± SD (mm Hg)	148.38 ± 25.06	146.80 ± 25.33	149.0 ± 25.5	0.835
DBP, mean ± SD (mm Hg)	82.32 ± 16.31	80.2 ± 12.0	83.2 ± 18.0	0.97
Stroke severity				
NIHSS at admission, median (IQR)	14 (8–19)	14 (8–19)	13.5 (8–19.5)	0.589
Large vessel occlusion location, *n* (%)				
Internal carotid artery	12 (35.3)	3 (30)	9 (37.5)	1
MCA	19 (55.9)	6 (60)	13 (54.2)	1
MCA, M1	16 (47.1)	5 (50)	11 (45.8)	1
MCA, Proximal M2	4 (11.8)	1 (10)	3 (12.5)	1
Anterior cerebral artery	3 (8.8)	0 (0)	3 (12.5)	0.539
Basilar artery	6 (17.6)	3 (30)	3 (12.5)	0.328
Posterior cerebral artery	3 (8.8)	1 (10)	2 (8.3)	1
TOAST etiology, *n* (%)				
Large artery atherosclerosis	10 (29.4)	3 (30)	7 (29.2)	1
Cardiac embolism	12 (35.3)	4 (40)	8 (33.3)	0.714
Cervical artery dissection	4 (11.8)	2 (20)	2 (8.3)	0.564
More than one etiology	2 (5.9)	1 (10)	1 (4.2)	0.508
Other determined etiology	2 (5.9)	1 (10)	2 (8.3)	1
Incomplete evaluation	4 (11.8)	0 (0)	4 (16.7)	0.296
Time of symptom onset (or last proof of good health) to groin puncture, median (IQR) (min)	257 (181–481)	285 (165–1084)	250 (193–448)	0.985
Wake-up stroke	4 (11.8)	1 (10)	3 (12.5)	1
Type of treatment, *n* (%)				
IV thrombolysis and EVT	8 (23.5)	2 (20)	6 (25)	1
Direct EVT	26 (76.5)	8 (80)	18 (75)	1
EVT success rate/TICI 2b/3	32 (94.1)	9 (90)	23 (95.8)	0.508
Clinical course, *n* (%)				
Symptomatic ICH	4 (11.8)	1 (10)	3 (12.5)	1
Recurrent stroke	1 (2.9)	0 (0)	1 (4.2)	1
Death in hospital	7 (20.6)	0 (0)	7 (29.2)	0.078

Categorical variables are compared using Fisher’s exact test, and continuous/ordinal variables using Mann–Whitney *U*-test

DBP, diastolic blood pressure; EVT, endovascular thrombectomy; ICH, intracerebral hemorrhage; IQR, interquartile range; MCA, middle cerebral artery; mRS, modified Rankin Scale; NIHSS, National Institutes of Health Stroke Scale; TIA, transient ischemic attack; TICI, thrombolysis in cerebral ischemia; TOAST, The Trial of Org 101072 in Acute Stroke Treatment

Overall, 10 (29.4%) achieved a favorable and 24 (70.6%) an unfavorable outcome at 3 months. Patient characteristics were comparable across both groups, especially regarding stroke severity according to NIHSS, LVO location, cardiovascular risk factors (excluding hypertension), and occurrence of symptomatic ICH (defined as a new hemorrhage on neuroimaging with a secondary NIHSS deterioration). Younger age (*p* = 0.025) and history of hypertension (*p* = 0.034), however, were more common in the group with favorable functional outcome.

### BPV

**Fig. 1:**
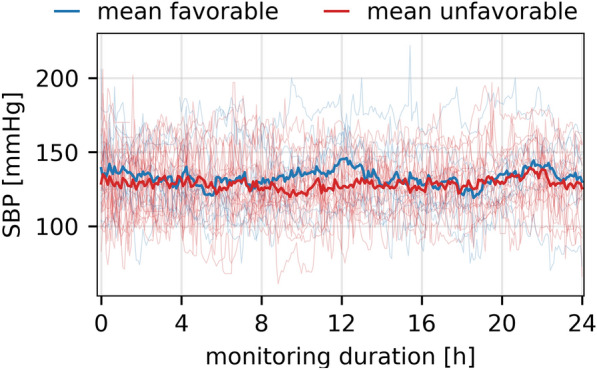
Raw SBP traces. Raw SBP traces of all patients with favorable (blue) or unfavorable (red) outcome over the monitoring duration of 24 h. SBP, systolic blood pressure

From the total of 34 patients, 23 patients had a complete 24-h BP record, 5 had a record between 12 and 24 h, and 5 had a record of less than 12 h (median 24.0 h, IQR 20.5–24.0 h). In the main article, we only present results for SBP, as no indices or results from spectral analysis were found to be significantly associated with outcome when using DBP (see Supplement Table 1) or MAP (data not shown). Mean absolute SBP (130 ± 18 mm Hg, *p* = 0.82; Fig. 1) as well as mean DBP (59 ± 10 mm Hg, *p* = 1.00) did not differ between the groups during the monitoring time.

### Indices and Time Domain

**Fig. 2:**
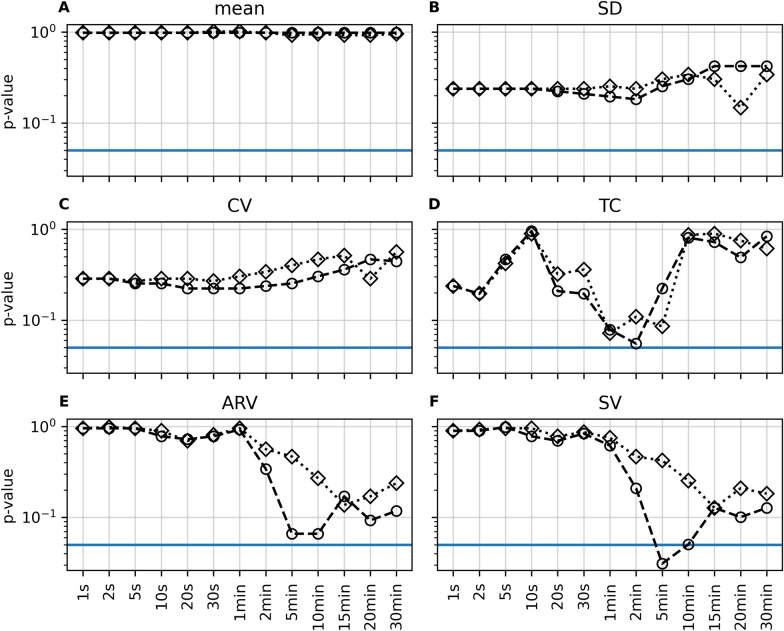
Mean SBP values and different systolic BPV indices in dependence of sampling frequency and downsampling methods (instantaneous sampling method and averaging method). For each index, the two curves show the *p* value to differentiate between the outcome groups using downsampling with either the instantaneous sampling method (diamond) or the averaging method (circle). The sampling frequency is given on the *x*-axis. The *y*-axis shows the respective *p* value of the Mann–Whitney *U*-test. The blue horizontal line indicates a *p* value of 0.05. ARV, averaged real variability, BPV, blood pressure variability, CV, coefficient of variation, SBP, systolic blood pressure, SD, standard deviation, SV, successive variation, TC, relative number of trend changes

Figure 2 depicts the *p* values for the various SBP BPV indices using different sampling intervals and the two downsampling methods (instantaneous sampling method and averaging method) for the outcome groups. The exact values for BPV of each index, based on its optimal sampling frequency (lowest *p* value), are presented in Table 2. BPV only reached predictive significance using SV extracted from downsampled (average over 5 min) SBP data (median 4.8 mm Hg [3.8, 7.1] vs. 7.1 mm Hg [5.5, 9.7], *p* = 0.031). The effect size found for SV was 0.74 (area under the curve, CI 0.57–0.85).

**Table 2:** SBP BPV indices and power of frequency bands

Parameter	*T*	Total	Favorable	Unfavorable	*p* value
SBP BPV indices (mm Hg)					
Mean	5 min	127.7 (122.1–139.8)	126.1 (121.5–147.1)	129.1 (123.4–137.4)	0.985
SD	2 min	12.63 (10.28–16.51)	11.95 (9.78–14.13)	13.27 (10.92–17.61)	0.183
CV	1 min	0.103 (0.076–0.127)	0.103 (0.063–0.109)	0.101 (0.086–0.141)	0.223
TC	2 min	0.442 (0.413–0.487)	0.489 (0.446–0.506)	0.432 (0.401–0.475)	0.055
ARV	5 min	4.517 (3.423–6.097)	3.498 (3.026–5.103)	5.146 (3.762–6.663)	0.066
SV	5 min	6.295 (4.742–8.614)	4.768 (3.804–7.070)	7.083 (5.506–9.709)	0.031
SBP power of frequency bands (mm Hg)^2^					
< 1/20 min	1 s	67.57 (44.74–122.02)	63.35 (41.81–82.02)	73.68 (48.59–139.29)	0.322
1/20 min to 1/5 min	1 s	11.42 (6.93–19.11)	6.76 (5.64–10.91)	12.59 (8.90–21.87)	0.020
1/5 min to 1/1 min	1 s	4.646 (3.983–7.166)	4.498 (4.067–8.343)	4.927 (3.722–6.929)	0.926
> 1/1 min	1 s	1.722 (1.314–2.569)	1.586 (1.308–6.161)	1.863 (1.363–2.563)	0.897

ARV, averaged real variability; BPV, blood pressure variability; CV, coefficient of variation; SBP, systolic blood pressure; SD, standard deviation; SV, successive variation; TC, relative number of trend changes; T, time bin

### Power Spectrum

Four different frequency bands were analyzed to evaluate BPV. Exact values of the power of the frequency bands can be found in Table 2. Predictive significance was only reached when analyzing the power of the midrange frequency band ranging from 1/20 min to 1/5 min (*p* = 0.020). An effect size similar to the one for SV could be found for the midrange frequency band (area under the curve = 0.75 [CI 0.59–0.86], see Supplement Fig. 3).

**Fig. 3:**
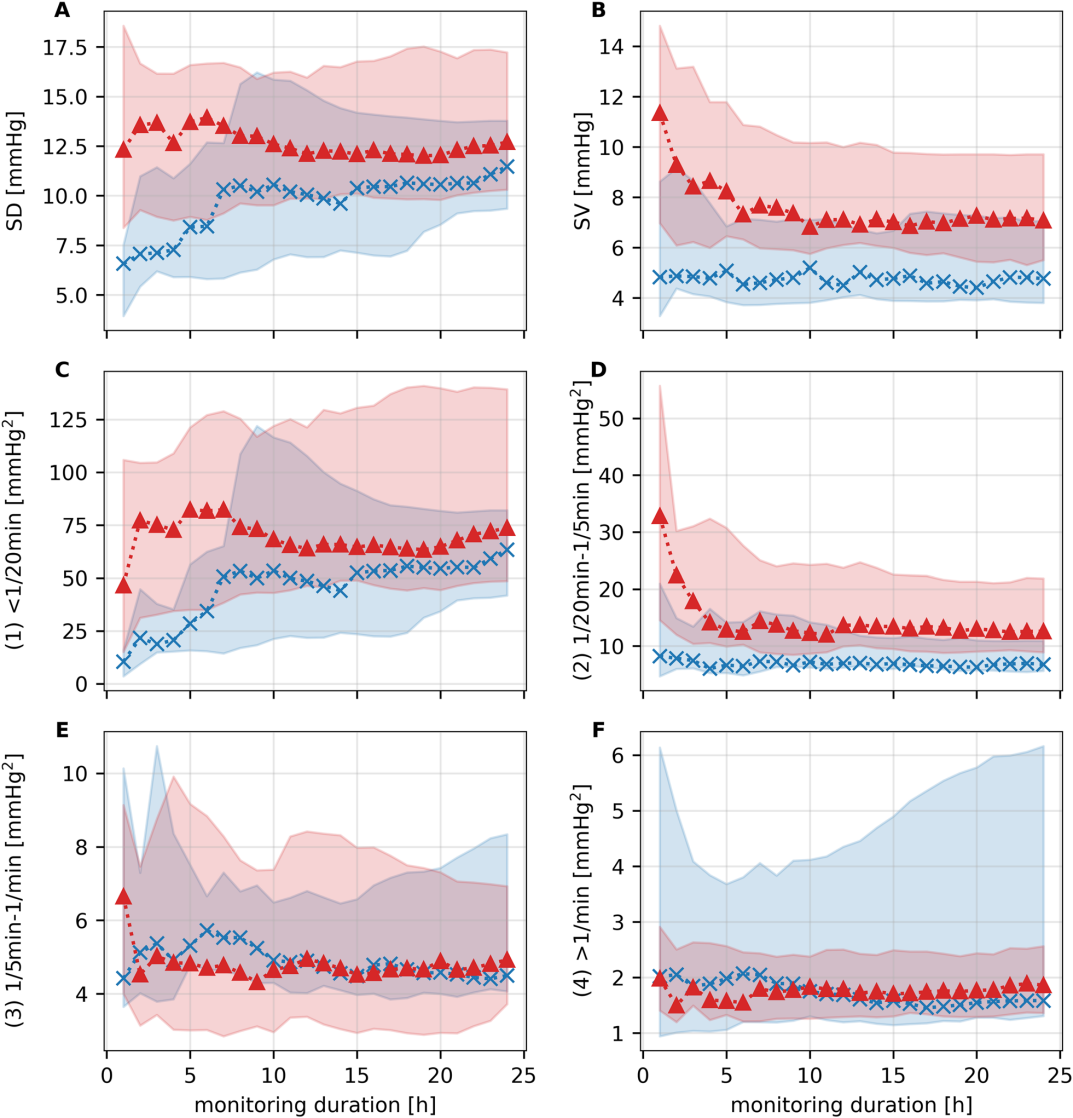
BPV indices and power of frequency bands depending on monitoring duration (raw data). Plots showing median SBP BPV values and interquartile ranges for SD, SV, and the power of frequency bands 1–4 for the two outcome groups (blue = favorable, red = unfavorable) computed for a continuously increasing monitoring duration. The interquartile ranges are represented by the shaded areas. BPV, blood pressure variability, SBP, systolic blood pressure, SD, standard deviation, SV, successive variation

### Monitoring Duration

**Fig. 4:**
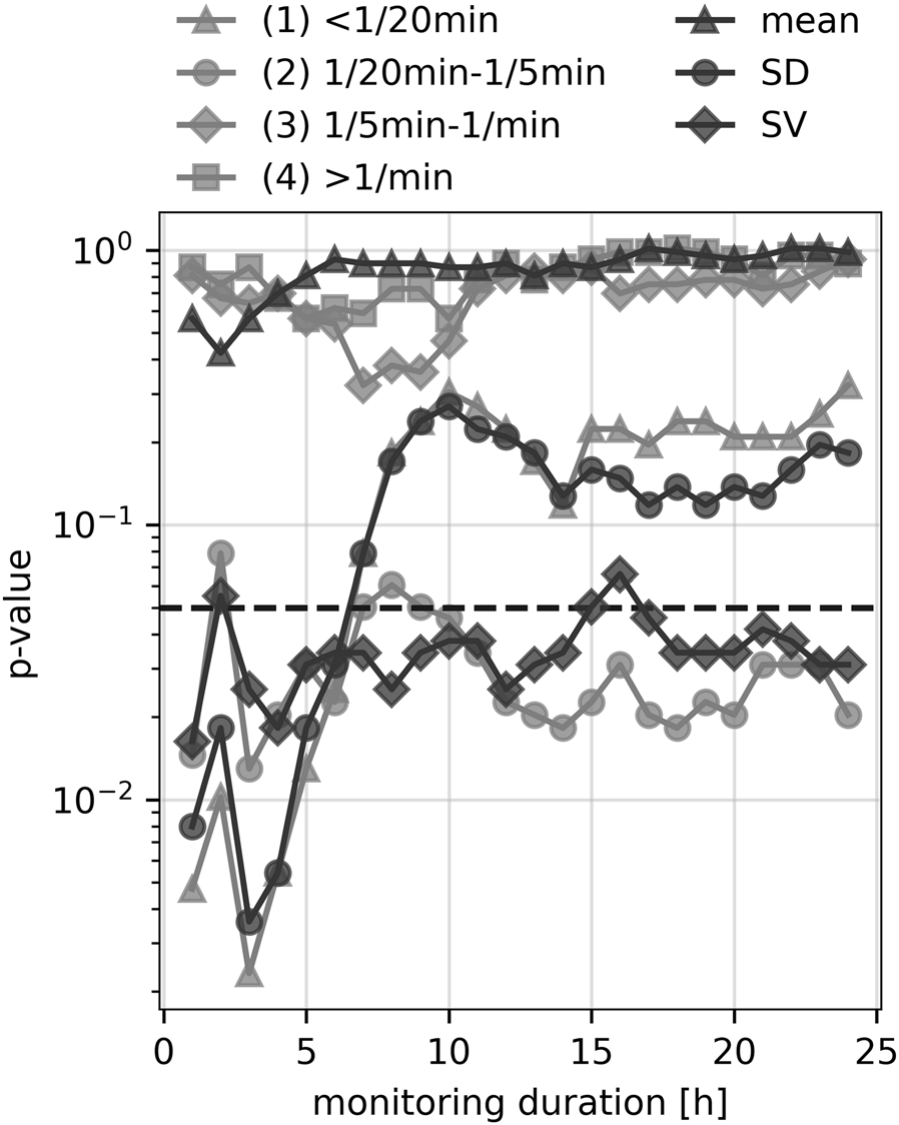
BPV indices and power of frequency bands depending on monitoring duration (*p* values). Semilogarithmic plot depicting *p* values of Mann–Whitney *U*-tests for BPV indices and power of frequency bands 1–4 computed for a continuously increasing monitoring duration. The dashed line indicates a *p* value of 0.05. BPV, blood pressure variability, SD, standard deviation, SV, successive variation

To investigate, whether earlier outcome prediction (before 24 h) is feasible, we computed BPV indices for monitoring durations starting with 1 h and incrementing up to the full duration of 24 h by hourly increases of included monitoring data (Figs. 3, 4).

For short monitoring durations up to 6 h, BPV, as evaluated using the SD or the power of low frequencies (< 1/20 min), was superior in distinguishing between the outcome groups in comparison to SV (Fig. 4). However, although SD and the power of low frequencies lost their predictive significance at around 6 h, SBP SV and the power spectrum of midrange frequencies (1/20 min to 1/5 min) remained predominantly unaffected by the monitoring duration (Fig. 4).

## Discussion

Our study was driven by the uncertainty of optimal BPV index and sampling frequency at which high-resolution BP data prove useful for outcome prediction following EVT in acute LVO stroke. Our results confirm previous reports that higher systolic BPV in the first 24 h is associated with unfavorable outcome [1, 6, 11]. In our study, systolic BPV acted as a stronger predictor of outcome than diastolic BPV, in accordance with previous reports [1, 4, 11, 12] and given the importance of SBP on penumbral blood flow [23].

SV reached predictive significance over the entire 24 h monitoring period if SBP recorded at 1 Hz was downsampled by averaging over 5-min intervals. However, none of the BPV indices showed a significant association if only instantaneous BP values were considered for BPV calculation. Contrarily, recent studies on patients with AIS after EVT reported a significant association while using sampling frequencies of up to 1/15 min in the first two hours and up to 1/30–60 min in the following 22 h, respectively [11, 24], likely due to a larger population size. Fittingly, SV and ARV showed a trend toward significance when lower sampling frequencies were used in our study. SV and ARV are known to be a more reliable representation of sequential variability and are less dependent on the absolute BP compared to SD and CV, whereas the last two only reflect the dispersion of BP measurements around the mean BP regardless of time series nature of the BP signal [25].

SV weighs frequencies close to the sampling frequency stronger than lower frequencies (see Supplement), thus measuring BPV in a sampling frequency dependent manner. As a result, SV is susceptible to aliasing stemming from undersampling. accordingly, SV computed from downsampled data using the averaging method performs better than just using instantaneous values because averaging counteracts aliasing by playing the role of a low-pass filter. SV in combination with the low-pass filter (downsampling using the averaging method) can then be interpreted as a measure for the variability in a sampling frequency dependent frequency band.

This theory is supported by the spectral approach that revealed the predictive value of the power of midrange frequencies (between 1/20 and 1/5 min)*.* The spectral analysis confirms that high frequencies components of the BP values hold no valuable information (mainly a source of noise) and low frequencies to be negligible for outcome prediction. Potential sources of noise in the measurement of BP in clinical settings might be change in head position, arm position, physical condition like distress, pain, and fever (e.g., shivering) [26]. Low frequencies, on the other hand, are likely due to diurnal BP variation [27]. On the other hand, the spectral analysis provided the ideal sampling rate for downsampling before SV calculation when using high-resolution SBP data. To portray the most relevant midrange frequencies found within the power analysis (between 1/20 and 1/5 min), a sampling rate of 2.5 min to 10 min is required using SV (the Nyquist-Limit) [19, 21].

Older studies on power spectrum analysis for evaluation of BPV attempted to provide insight into its physiological and clinical interpretations to study the mechanisms involved in cardiovascular regulation. Sympathetic vascular and cardiac modulation appear to be reflected best by BP frequencies around 0.1 Hz. In rats, modulation by the renin-angiotensin system, catecholamines, and shear stress-induced release of nitric oxide (from the endothelium and the myogenic vascular response), affect lower BP frequencies of 0.02–0.2 Hz, whereas effects in humans remains inconclusive [20, 28].

To further elucidate the importance of monitoring duration, we analyzed a continuously increasing monitoring duration starting from 1 h (increasing by steps of 1 h). Here we obtained a surprising result. Within the first few hours, not only SV and midrange frequency power, but also SD and low-frequency power were predictive. However, only SV and midrange frequency power remained significant over the entire monitoring period of 24 h. Reasoning for this result is speculative. It is possible that slow trends in total BP values dominated SD and low-frequency power during the first few hours. However, as absolute BP became more stable over time, this contribution decreased with increasing duration of monitoring.

Mean absolute SBP did not differ between outcome groups in our setting, thereby further emphasizing the importance of BPV in stroke management in line with growing knowledge [4, 11]. Considering all stroke etiologies, the optimal hemodynamic management after AIS is still to be determined. Lowering absolute SBP only reduces occurrence of ICH but does not lead to improved functional outcome [29, 30]. In contrast, Goyal et al. [18] recently demonstrated that achieving a BP of < 160/90 mm Hg during the first 24 h following EVT was independently associated with a lower 3-month mortality and favorable functional outcome. Given the conflicting data on the optimal BP target, in recent years, more focus has been put on BPV, as sustained and smoothened BP might enhance outcome.

There are several limitations. First, the number of patients (total of 34) is small and the study is retrospective in nature. These factors come with known drawbacks leading to a potentially low external validity. Second, there is a significant selection bias. Patients are commonly extubated after EVT. Thus, generally only more severe cases are transferred to the ICU (as can be deduced from the predominance of unfavorable outcome). Patients who reach a favorable outcome are less likely to be referred to the ICU, making our data set unbalanced. Third, the presence of cardiovascular risk factors, such as, in our case, hypertension in young patients, might have led to the initial decision to monitor these patients in the ICU rather than the severity of the disease itself. Fourth, 30% vs. 37.5% of patients in either group had atrial fibrillation. These might have altered the BPV. However, excluding these patients would have significantly reduced the future applicability of these results. Furthermore, the manual exclusion of episodes with atrial fibrillation would inhibit potential straightforward implementation. Fifth, power spectral analyses are susceptible to artifacts in raw or preprocessed data. We opted to keep preprocessing of our data to a minimum, as any alteration of the raw data can also lead to unintended bias. Data stability could be confirmed by the agreement of the SV index observed with the frequency domain analysis. Therefore, the simple threshold filter used successfully removed artifacts without suppressing the inherent physiological variability of the BP. Sixth, there is conflicting evidence of the effect of recanalization status on the predictive significance of BPV. Although some studies only find an effect of BPV in patients without successful recanalization [1, 24, 31], others find the exact opposite result [6, 11]. In our population, 94% of the occluded vessels were successfully recanalized, which made an analysis based on revascularization status impossible. Seventh, only 23 out of 34 patients had a complete 24-h BP record. However, BPV indices reached predictive significance within the first few hours that included data from all patients. Thus, further data (after the first few hours) were not imperative for the result. Eighth, there is a variety of other factors that could have influenced outcome or BPV itself, especially comedication or clinical and baseline characteristics: these include (among others) the use of analgosedatives and antihypertensive medication as well as catecholamines, renal dysfunction, possible cardiopulmonary complications related to systemic hemodynamics, intracranial hemorrhage, recurrent stroke, and the relative change in BP from baseline at presentation. However, because of the small sample size, we decided against correcting for confounders. Lastly, because of the novelty of data (high-resolution BP data) and some of the indices used, no sample size calculation was available.

## Conclusions

Our results show that the use of high-resolution BP data can lead to more predictive BPV indices. When using high-resolution BP data, downsampling (using a moving average over 5 min) might be essential to find relevant changes in systolic BPV, as noise can be adequately suppressed (as confirmed by the significance of the power of midrange frequencies). Furthermore, we show that SV and the power of midrange frequencies provide consistent results over a wide range of measurement durations, making these promising for future real-world outcome prediction. Although SV as well as the power of midrange frequencies are similar in origin, in clinical practice, SV might be easier to implement because of its simple mathematical function. Further large-scale studies will be necessary to validate these results. Furthermore, whether increased BPV is the cause for unfavorable outcome, just an expression of the severity of disease, or even malleable by medical intervention remains to be determined.

This study provides a detailed description of the possibilities and merits of using high-resolution BP data in BPV indices calculation for outcome prediction in AIS-LVO. However, to corroborate the extracted hypothesis a prospectively designed study with a clear endpoint, appropriate sample size calculation (based on the data presented) along with adjustment for confounding factors is necessary to fully understand the role of BPV in patients with AIS-LVO.

## Supplementary Information

Below is the link to the electronic supplementary material.

Supplementary file1 (DOCX 4317 kb)
