# Supplementary material for: Blood Pressure Variability Indices for Outcome Prediction After Thrombectomy in Stroke by Using High-Resolution Data
Source: Neurocrit Care. 2022 May 23;37(Suppl 2):220–9. doi: 10.1007/s12028-022-01519-x (PMC9343264; doi:10.1007/s12028-022-01519-x)
Supplement: Supplementary file 1 — Supplementary file1 (DOCX 4317 kb) [file 12028_2022_1519_MOESM1_ESM.docx]

**SUPPLEMENTAL MATERIAL**

**Supplemental Methods**

**Frequency Dependence of the SV Index**

In the manuscript, we note that the SV is more representative for the variability at higher frequencies, i.e., the frequencies closer to the Nyquist frequency. This property of the SV can be understood looking at the following approximation (for n>>1), which relates the square of the SV to a weighted sum over the power spectrum:

$$\left( SV \right)^{2}=\frac{1}{n-1}\sum_{i=1}^{n-1} \left| BP_{i+1}-BP_{i} \right|^{2}\approx\frac{1}{n^{2}}\sum_{k=0}^{n-1} \left| \hat{BP_{k}} \right|^{2}\left( 1-\cos\left( \frac{2\pi k}{n} \right) \right),$$

where $\hat{BP_{k}}$ are the Fourier components of the blood pressure signal. The right-hand-side of the approximation shows how the Fourier components are weighted depending on their frequency. For example, let k=0, then the cosine is equal to one and thus the corresponding weight exactly zero. For the other extreme, let k=n/2, then the cosine is (-1) and the weight is equal to 2. In contrast, the total signal power, which is given by the variance of the signal, exhibits no such frequency dependence:

$$\left( SD \right)^{2}\approx\frac{1}{n^{2}}\sum_{k=0}^{n-1} \left| \hat{BP_{k}} \right|^{2},$$

where the approximation holds as 1/($n-1)\approx1/n$ for large n.

Figure I illustrates this behavior for an artificial signal consisting of only two frequency components. The two frequency components have the same amplitude, nevertheless their contribution to the SV will be very different as one is close to the Nyquist frequency and the other far from it. The contribution of the frequency component far from Nyquist frequency gets clearly suppressed.

**Frequency Bands Used in Frequency Domain Analyses**

We introduced four frequency domain indices that summarize the power of four distinct frequency bands. The location of the mid-range band was thereby motivated by the behavior of the SV when applied to blood pressure signals resampled at different sampling rates. Table II lists the frequency range of each frequency band, and Figure II depicts a typical blood pressure signal as well as its corresponding power spectral density, and the frequency bands.

**Correlation Between SV and Power of Frequency Band**

As mentioned above, the band power of the mid-range frequencies (frequency band 2) was motivated by the behavior of the SV computed from down-sampled SBP signals at different sampling times. As described in the main text, we found that when a low-pass filter was applied before resampling the signal, the SV performed best for a sampling time of 5 min.

The sampling time could then be related to a frequency range using the frequency dependence of the SV. To do so, we first note that for a sampling time of 5 min, the Nyquist frequency is 1/10min. It follows that the variability mesured by the SV stems predominantly from the frequency range 1/10min to 1/20min, as slower frequency will be strongly suppressed by the SV index’s particular frequency dependence.

However, since the p-value for the SV starts already dropping somewhere between a sampling time of 2 min and 5 min, we defined the mid-range frequency band as the range between 1/5min and 1/20min.

Importantly, frequencies higher than 1/5min were suppressed by the low-pass filter used in the down-sampling procedure. Figure III confirms the connection between the SV and the mid-range power band by comparing their values and their receiver operating characteristics (ROC).

**Remarks on Aliasing**

In time series analysis, aliasing is an effect that occurs when signals are sampled at a rate less than twice their true frequency and it can result in spectra with displaced frequency components (frequency components appear at wrong frequencies in the spectrum). Often aliasing makes it impossible to correctly assign apparent frequency components to their true frequencies (unless using specialized compressed sensing techniques).

Thus, whenever possible, sampling rates above the Nyquist-Limit (f_s > 2*f_signal) should be used to digitalize signal to prevent aliasing. Figure IV illustrates the aliasing effect of a simulated signal.

Figure IV illustrates, how the original signal at a frequency of 1/(5min) appears at a much lower frequency of about 1/(20min) in the spectrum computed from the under-sampled signal (orange). In fact, it can be seen from the lower right subplot, how the peak in the spectrum gets mirrored on a vertical line at the position of the Nyquist frequency (f_s/2).

Importantly, aliasing does not change the power in the frequency components. Thus, variability indices that weight all frequency components equally, or do not measure power at all will be unaffected. This means that for example the mean or the SD are indices that are robust with respect to under-sampling (i.e. taking blood pressure measurements at a lower rate). The SV, on the other hand, mainly measures the power in the higher frequency components, which means frequencies close to the Nyquist frequency (f_s/2). Thus, the performance of the SV is expected to vary when changing the sampling frequency, which is exactly what we found in our analysis.

Moreover, if high-resolution blood pressure data is available aliasing can be avoided by applying a low-pass filter before down-sampling the signal. In our analysis, we considered both scenarios and found that low-pass filtering is crucial for the computation of a predictive SV index. This clearly emphasizes the advantage of having access to high-resolution data.

**Supplemental Tables**

**Table I. DBP BPV Indices and Power of Frequency Bands**

|  | **unit** | **t** | **total** | **favorable** | **unfavorable** | **p-value** |
| --- | --- | --- | --- | --- | --- | --- |
| **DBP BPV Indices** |  |  |  |  |  |  |
| mean | mmHg | 5min | 57.7 [51.9, 63.6] | 60.0 [51.9, 67.7] | 56.5 [53.0, 62.8] | 0.809 |
| SD | mmHg | 2min | 6.41 [5.53, 7.83] | 5.98 [5.38, 7.32] | 6.46 [5.68, 7.99] | 0.381 |
| CV |  | 1min | 0.111 [0.090, 0.148] | 0.106 [0.082, 0.143] | 0.114 [0.095, 0.146] | 0.341 |
| TC |  | 2min | 0.470 [0.441, 0.490] | 0.483 [0.470, 0.501] | 0.454 [0.431, 0.482] | 0.093 |
| ARV | mmHg | 5min | 2.223 [1.750, 3.081] | 1.826 [1.711, 2.560] | 2.644 [1.903, 3.248] | 0.109 |
| SV | mmHg | 5min | 3.308 [2.566, 4.483] | 2.613 [2.339, 3.746] | 3.661 [2.874, 4.720] | 0.101 |
| **DBP Power of Frequency Bands** |  |  |  |  |  |  |
| <1/20min | (mmHg)^2^ | 1s | 17.82 [12.32, 26.75] | 15.50 [12.13, 22.66] | 18.56 [12.32, 28.91] | 0.445 |
| 1/20min to 1/5min | (mmHg)^2^ | 1s | 3.37 [2.19, 5.25] | 2.92 [1.80, 4.23] | 3.44 [2.64, 5.57] | 0.287 |
| 1/5min to 1/min | (mmHg)^2^ | 1s | 1.802 [1.413, 2.463] | 1.720 [1.598, 2.285] | 1.888 [1.380, 2.564] | 0.956 |
| > 1/min | (mmHg)^2^ | 1s | 0.926 [0.687, 1.075] | 1.012 [0.704, 1.330] | 0.857 [0.687, 0.984] | 0.445 |

ARV, averaged real variability; BPV, blood pressure variability; CV, coefficient of variation; DBP, diastolic blood pressure; SD, standard deviation; SV, successive variation; TC, relative number of trend changes; T, time bin

**Table II. Definitions of Frequency Bands**

|  | Start frequency | Stop frequency |
| --- | --- | --- |
| Frequency Band 1 | 0/min | 1/(20 min) |
| Frequency Band 2 | 1/(20 min) | 1/(5 min) |
| Frequency Band 3 | 1/(5 min) | 1/min |
| Frequency Band 4 | 1/min | 30/min |

**Supplemental Figures and Figure Legends**

**Figure I. Frequency Domain Representation of the SV Index.**


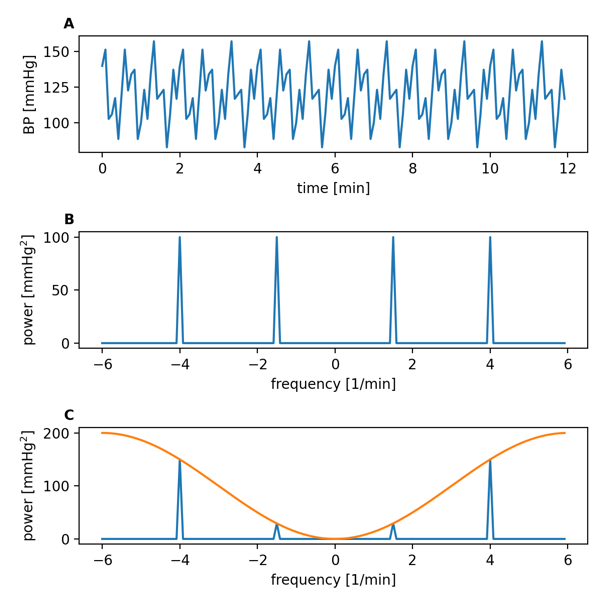


Plot **A** and **B** depict an artificial signal with two frequency components (f_1=1.5/min, f_2=4/min) and its power spectrum, respectively. The signal is sampled at a frequency of 12/min (0.2 Hz) and was detrended before computing the power spectrum. Plot **C** illustrates the frequency response of the SV index in orange and the weighted frequency components in blue for the signal depicted in **A**.

**Figure II. Illustration of Frequency bands.**


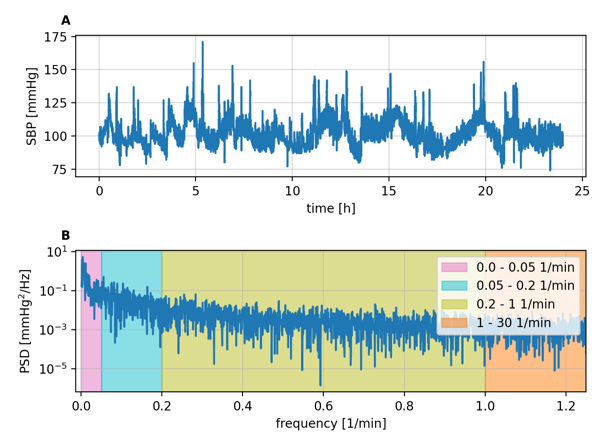


Plot **A** shows a SBP signal measured at 1 Hz for the first 24 hours of the patient’s stay in the ICU. The depicted SBP signal has been preprocessed to remove artifacts. Plot **B** shows the derived power spectral density of the same SBP signal cropped at a frequency of 1.25/min for illustration purposes. The different frequency bands are indicated by differently colored backgrounds.

**Figure III. Comparison between SV index and mid-range frequency band.**

**
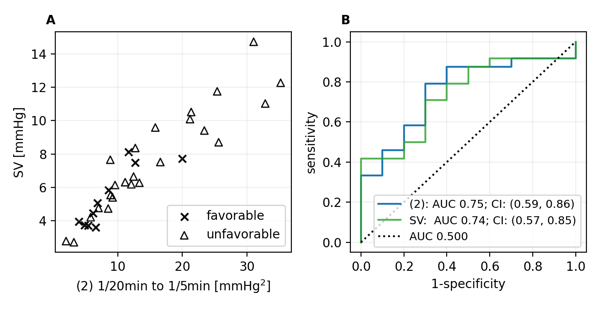
**

Plot **A** depicts a scatter plot with the SV index on the y-axis and the value of the power in the mid-range frequency band on the x-axis. Plot **B** shows the ROC and states area under the curve ^17^ in the legend. The SV index is drawn in green and the power of the mid-range frequencies (frequency band 2) in blue. The confidence intervals (CI) in the legend were computed using Newcombe’s method.

**Figure IV. Aliasing Effect of a Simulated Signal.**

**
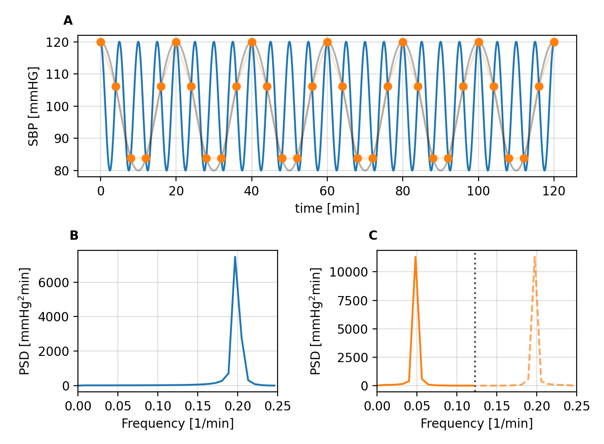
**

Plot **A** depicts in blue the true signal comprising a single frequency component at f_0=1/(5min) and in orange the under-sampled signal using a sampling frequency of f_s=1/(4 min). Plots **B** and **C** show the corresponding spectra in matching colors (solid lines). For the blue spectrum, we used a sampling rate of 1/(2min), which is above the Nyquist-Limit. Furthermore, to illustrate how aliasing folds the true spectrum, we also depict the unfolded spectrum for the under-sampled signal (orange dashed line).
